# Toxin Profiling of *Amanita citrina* and *A. sinocitrina*: First Report of Bufotenine Detection

**DOI:** 10.3390/toxins17050247

**Published:** 2025-05-16

**Authors:** Yi-Zhe Zhang, Yi Yao, Kai-Ping Zhang, Jia-Qi Liang, Jia-Ju Zhong, Zhong-Feng Li, Hai-Jiao Li, Fei Xu

**Affiliations:** 1 State Key Laboratory of Trauma and Chemical Poisoning, National Institute of Occupational Health and Poison Control, Chinese Centre for Disease Control and Prevention, No. 29, Nanwei Road, Xicheng District, Beijing 100050, China; zyz97@263.net (Y.-Z.Z.); zhongjiaju1990@126.com (J.-J.Z.); i1175cayy@163.com (Z.-F.L.); 2 School of Public Health, Ningxia Key Laboratory of Environmental Factors and Chronic Diseases Control, Ningxia Medical University, No. 1160, Shengli South Road, Xingqing District, Yinchuan 750004, China; yyi25168@163.com; 3 Zichuan District National Forest Farm, Zibo 255100, China; zhangkaiping1995@126.com; 4 Center for Disease Control and Prevention of Yiyang, Yiyang 413000, China; ljq971202@163.com; 5 Physical and Chemical Department, Ningxia Hui Autonomous Region Center for Disease Control and Prevention, Yinchuan 750004, China

**Keywords:** *Amanita*, poisonous mushroom, species identification, toxin detection, tryptamine alkaloids

## Abstract

*Amanita* species are widely distributed worldwide. Many of these species are poisonous and can cause health problems, resulting in morbidity and mortality. The toxins responsible for poisoning are amatoxins, aminohexadienoic acid, ibotenic acid, muscimol and muscarines, which damage the liver, kidney, central nervous system and parasympathetic nervous system. In recent years, several toxins have been discovered from different poisonous mushrooms. In this study, multiwalled carbon nanotube purification and ultrahigh-performance liquid chromatography–tandem mass spectrometry (UPLC-MS/MS) was used for the sensitive detection and targeted quantitative screening of 12 mushroom toxins (muscarine, two isoxazole derivatives, three tryptamine alkaloids, three amatoxins and three phallotoxins) from *Amanita citrina*, *A. citrina* var. *grisea* and *A. sinocitrina*. This study found that bufotenine, one of the tryptamine alkaloids, was detected in *A*. *citrina* and *A*. *sinocitrina* with an average content of 2.90 and 1.19–6.70 g/kg (*n* = 3) in the dried mushrooms, respectively. None of the 12 common toxins were discovered in *A*. *citrina* var. *grisea*. These results provide reference data for future research on the role of toxins in the evolution of *Amanita* mushrooms. Future studies should explore the biosynthetic pathways and ecological roles of these toxins in *Amanita* species.

## 1. Introduction

*Amanita* Pers. species are economically and ecologically important, with >700 accepted species worldwide [1,2,3,4,5,6,7,8,9,10,11,12,13,14,15,16,17,18,19,20,21,22,23,24,25,26,27,28,29,30,31,32,33,34,35,36,37,38,39,40]. Based on recent comprehensive phylogenetic studies, *Amanita* is divided into three subgenera (*Amanita*, *Amanitina* and *Lepidella*) and eleven sections [16,24,39,41]. Interestingly, the species edibility is more or less similar in the different sections [18,19,24,42].

Many *Amanita* species are famous edible species. For example, *Amanita caesarea* (Scop.) Pers. from *Amanita* sect. *Caesareae* is a common delicious wild mushroom in Europe [20,43,44]. Most species of *Amanita* sect. *Caesareae* and some species of sect. *Roanokenses* and sect. *Amanita* in China are edible and are widely collected and consumed, such as *A. caojizong* Zhu L. Yang; Yang-Yang Cui & Qing Cai; *A. caesareoides* Lj. N. Vassiljeva; *A. hemibapha* (Berk. & Broome) Sacc.; *A. rubromarginata* Har. Takah.; *A. kitamagotake* N. Endo & A. Yamada; *A. ochracea* (Zhu L. Yang) Yang-Yang Cui, Qing Cai & Zhu L. Yang; *A. princeps* Corner & Bas; *A. pseudoprinceps* Yang-Yang Cui, Qing Cai & Zhu L. Yang; *A. rubroflava* Yang-Yang Cui, Qing Cai & Zhu L. Yang; *A. subhemibapha* Zhu L. Yang, Yang-Yang Cui & Qing Cai and *A. sinensis* Zhu L. Yang [18,19,24,45].

In contrast, many *Amanita* species are poisonous or even deadly, resulting in acute liver and renal failure and psychoneurological disorders [18,19,24,42,46,47,48,49,50,51,52,53,54,55,56,57,58]. The most notorious lethal *Amanita* are from *Amanita* sect. *Phalloideae*, with >70 species producing cyclic peptide toxins, responsible for >70% of all mushroom poisoning fatalities [42,49,50,51,52,53,54,55,56,57,58,59]. Cyclic peptide toxins are classified into three major groups: amatoxins, hallotoxins and virotoxins, with 39 toxins reported [59,60,61,62,63,64,65,66,67,68,69]. The most famous lethal *Amanita* species in Europe and North America include *A. phalloides* (Vaill. ex Fr.) Link, *A. verna* Bull. ex Lam., *A. virosa* Bertill., *A. ocreata* Peck, *A. bisporigera* G.F. Atk., *A. suballiacea* (Murrill) Murrill and *A. tenuifolia* (Murrill) Murrill [60,64,70,71,72]. In China, 12 deadly *Amanita* have been reported, and the most common species in mushroom poisoning outbreaks include *A. exitialis* Zhu L. Yang & T. H. Li, *A. fuliginea* Hongo, *A. rimosa* P. Zhang & Zhu L. Yang, *A. subjunquillea* S. Imai, *A. pallidorosea* P. Zhang & Zhu L. Yang, *A. subpallidorosea* Hai J. Li, *A. fuligineoides* P. Zhang & Zhu L. Yang and *A. subfuliginea* Q. Cai, Zhu L. Yang & Y.-Y. Cui [42,52,73,74,75,76].

Thirteen species of mushroom, including *A. abrupta* Peck, *A. boudieri* Barla, *A. echinocephala* (Vittad.) Quél., *A. gracilior* Bas & Honrubia, *A. gymnopus* Corner & Bas, *A. kotohiraensis* Nagas. & Mitani, *A. nauseosa* (Wakef.) D.A. Reid, *A. neoovoidea* Hongo, *A. oberwinklerana* Zhu L. Yang & Yoshim. Doi, *A. ovoidea* (Bull.) Link, *A. proxima* Dumée, *A. pseudoporphyria* Hongo and *A. smithiana* Bas, produce aminohexadienoic acid (mainly 2-amino-4,5-hexadienoic acid) and cause acute renal failure [54,55,56,57,58,77,78,79,80,81,82,83,84,85]. In China, *A. oberwinklerana*, *A. pseudoporphyria*, *A. neoovoidea* and *A. gymnopus* are the most common species responsible for mushroom poisoning incidents [42,52,54,55,56,57,58,83,86].

Most species in *Amanita* sect. *Amanita* contain neuropsychiatric toxins, including isoxazole derivatives and muscarine [18,19,38,42,52,87]. More than 100 species of sect. *Amanita* have been reported worldwide ([http://www.amanitaceae.org] on 1 February 2025), and 28 taxa have been recognized in China [18,19,24,38,40]. In Europe and North America, *A. muscaria* (L.) Lam. and *A. pantherina* (DC.) Krombh. are the two most common species responsible for poisoning incidents. *A. aprica* J. Lindgr. & Tulloss, *A. gemmata* (Fr.) Bertill., *A. regalis* (Fr.) Michael and *A. ibotengutake* T. Oda et al. are also poisonous [87,88,89,90,91,92]. In China, >10 species, including *A. subglobosa* Zhu L. Yang; *A. sychnopyramis* f. *subannulata* Hongo; *A. concentrica* T. Oda, C. Tanaka & Tsuda; *A. melleiceps* Hongo; *A. parvipantherina* Zhu L. Yang, M. Weiss & Oberw.; *A. pseudosychnopyramis* Y.Y. Cui, Q. Cai & Zhu L. Yang; *A. rufoferruginea* Hongo; *A. siamensis* Sanmee, Zhu L. Yang, P. Lumyong & Lumyong; *A. ibotengutake*; *A. melleialba* Zhu L. Yang, Q. Cai & Y.Y. Cui; *A. orientigemmata* Zhu L. Yang & Yoshim. Doi; *A. pseudopantherina* Zhu L. Yang ex Y.Y. Cui, Q. Cai & Zhu L. Yang and *A. griseopantherina* Y.Y. Cui, Q. Cai & Zhu L. Yang, were discovered in mushroom poisoning incidents [42,52,54,55,56,57,58].

Some species of *Amanita* sect. *Validae* also exhibit neurotoxicity. *A. citrina* Pers. does not contain isoxazole derivatives and muscarine but produces bufotenine at a concentration of 1.6–7.5 mg/g dry weight [59,93,94,95]. *Amanita porphyria* Alb. & Schwein.: Fr. has been reported to contain 5-hydroxytryptophan [94].

*Amanita citrina* var. *grisea* (Hongo) Hongo was first described in Japan and was later reported in China [24,96,97,98]. In 2001, *A. sinocitrina*, a new species morphologically similar to *A. citrina*, was described in China [99]. Whether these two taxa also have similar toxins is a fascinating question. While *A. citrina* has been reported to contain bufotenine, the chemical profiles of var. grisea and *A. sinocitrina* remain unknown. To determine this, we conducted a study using ultrahigh-performance liquid chromatography–tandem mass spectrometry (UPLC–MS/MS) for toxin profiling and ITS sequencing for phylogenetic analysis, comparing the chemical and genetic diversity among the three species. Our findings showed that bufotenine, one of the tryptamine alkaloids, was detected in *A*. *citrina* and *A*. *sinocitrina*, and none of the 12 common toxins were discovered in *A. citrina* var. *grisea*. The detailed description of species identification and toxin analysis is shown in this study below.

## 2. Results

### 2.1. Mushroom Identification

**Figure 1:**
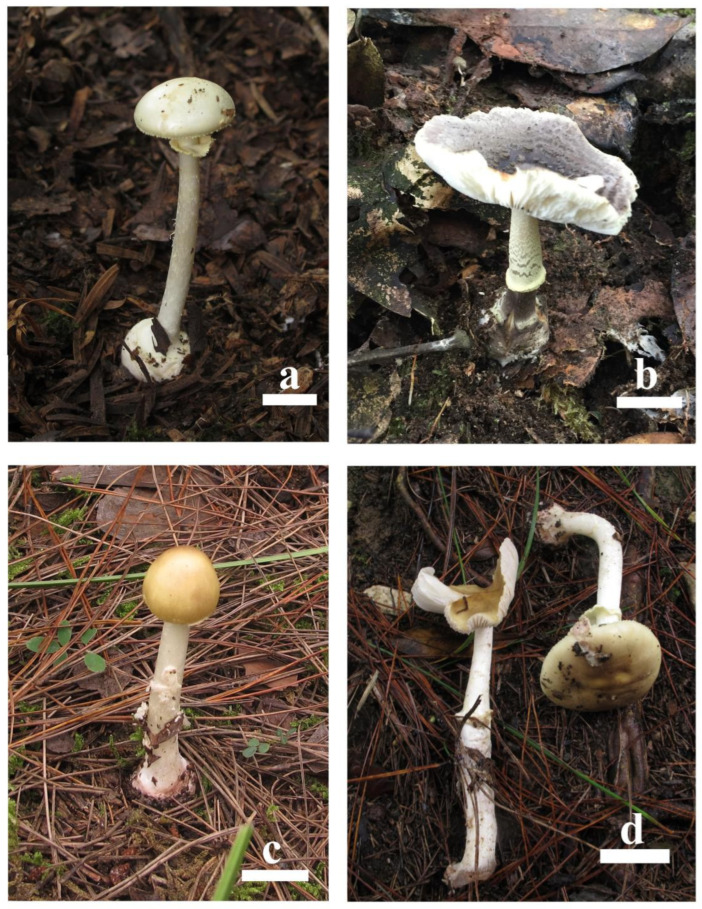
Basidiomata of *Amanita* species studied in the present paper: (**a**) *A. citrina* (150913-24), (**b**) *A. citrina* var. *grisea* (WX20170922-067), (**c**) *A. sinocitrina* (150914-18), (**d**) *A. sinocitrina* (171012-23). Bars = 1 cm.

**Figure 2:**
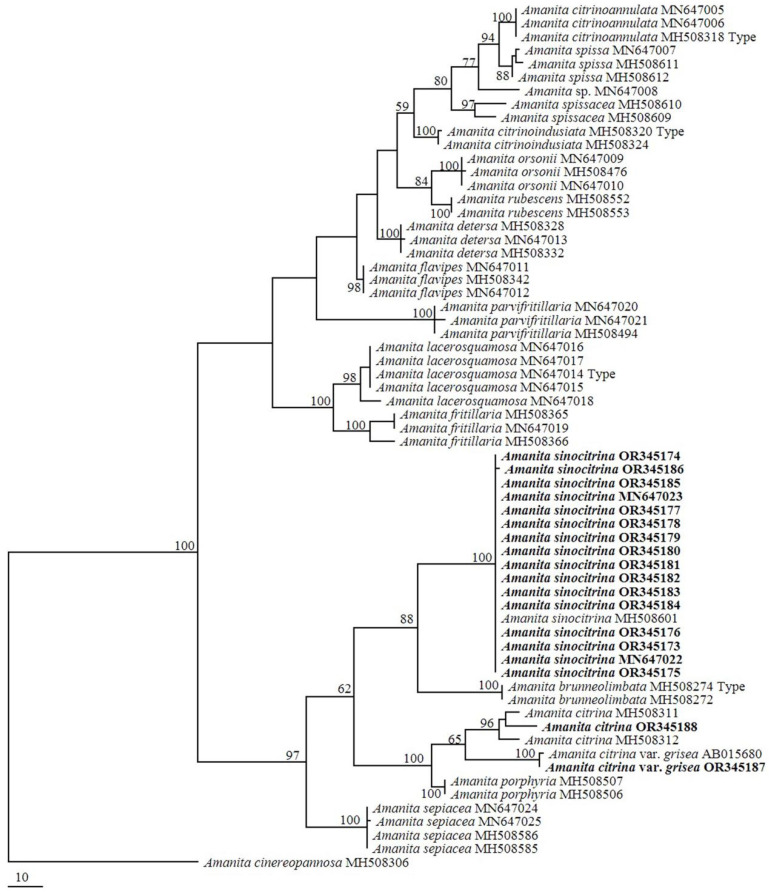
Phylogenetic tree inferred from MP analysis based on internal transcribed spacer (ITS) sequences. Only bootstrap values > 50% are reported. Sequences from present study are shown in bold.

Based on morphological and phylogenetic analyses, all 18 specimens were successfully identified, including 1 specimen of *Amanita citrina*, 1 specimen of *A. citrina* var. *grisea* and 16 specimens of *A. sinocitrina* (Table 1; Figure 1 and Figure 2).

### 2.2. Methodology Examination and Toxin Detection Results

**Figure 3:**
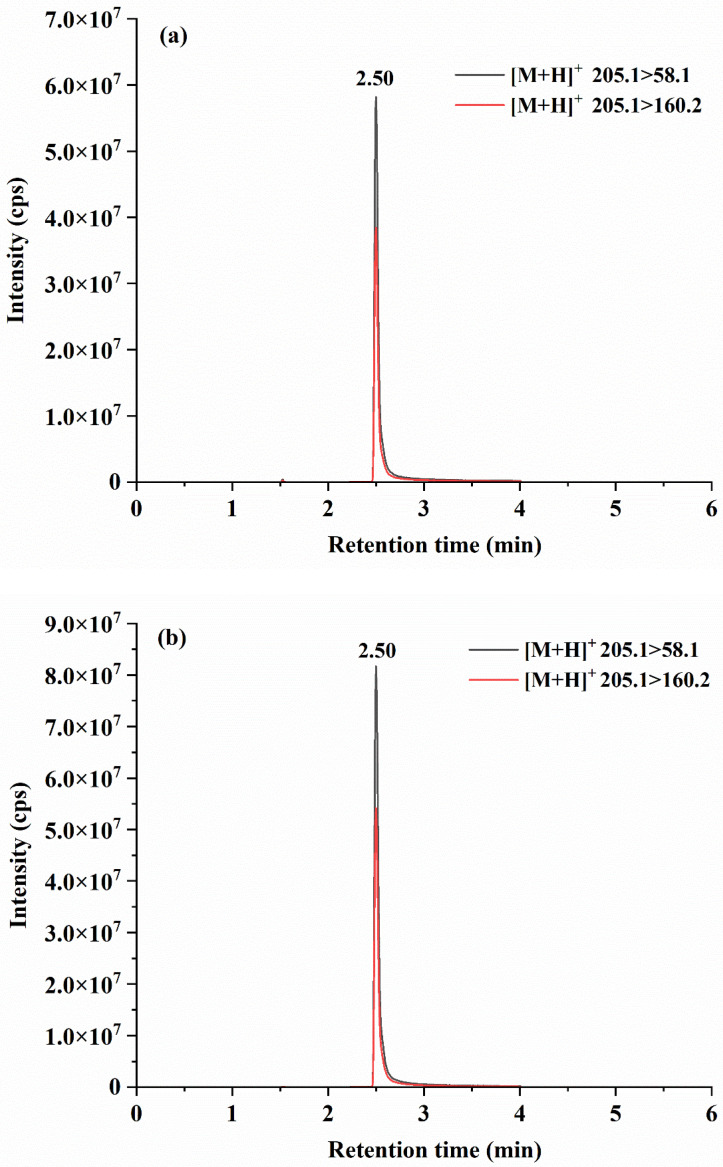
MRM chromatograms of bufotenine in *A. citrina* (**a**) and *A. sinocitrina* (**b**).

UPLC–MS/MS targeted screening results showed that bufotenine was detected in *A*. *citrina* and *A*. *sinocitrina*. None of the other 11 toxins, including muscarine, psilocybin, psilocin, ibotenic acid, muscimol, α-amanitin, β-amanitin, γ-amanitin, phallacidin, phallisacin and phalloidin, were detected (Figure 3). In addition, none of the toxins were detected in *A*. *citrina* var. *grisea*. In quantitative analysis, the limits of detection (S/N = 3) and limits of quantification (S/N = 10) of bufotenine in the matrix blank sample were 0.1 and 0.2 mg/kg, respectively. Bufotenine showed good linearity (*R*^2^ > 0.9960), the three levels of recoveries (10, 20 and 100 mg/kg) of bufotenine in spiked *L. edodes* samples were 92.3–96.5% and the relative standard deviations were 3.7–5.6%. This method has certain advantages in the extraction and purification of mushroom toxins. Statistical comparisons were made using one-way analysis of variance. UPLC–MS/MS analyses showed that the average contents of bufotenine were 2.90 ± 0.27 g/kg in *A. citrina* and ranged from 1.19 ± 0.02 to 6.70 ± 1.36 g/kg in *A. sinocitrina* (*n* = 3), respectively, in the Guizhou, Yunnan, Hubei and Hunan regions in China (Table 1).

## 3. Discussion

Bufotenine, a tryptamine alkaloid, was first described in the 1920s [100,101,102,103] and discovered in different living organisms, such as in the skin secretions of many toads (such as of the *Bufo* species) [104], plants of the Leguminosae family and fungi [59,103,104,105,106,107]. Bufotenine is present as a part of a complex skin secretion in almost 200 species of the genus *Bufo* worldwide; this secretion acts as a defense mechanism against predators and has antibacterial and antiviral properties [108]. Since the 1960s, there have been reports of smoking dried toad skin secretions, and since the 1980s, reports of “toad licking” have been increasing [109].

Structurally, bufotenine is very similar to psilocin (the active form of psilocybin), but differs in having a hydroxyl group on the C5 of the indole ring instead of C4 [59,103,107]. The possible hallucinogenic effects noted in the literature are still controversial; due to bufotenine not crossing the blood–brain barrier, it may lack hallucinogenic effects [107,110]. However, bufotenine is a promising candidate for treating rabies [107,111].

More than 100 species in *Amanita* sect. *Validae* have been discovered worldwide, and approximately 25 of these species were reported in China [18,19,24,39,40,100]. Previous studies showed that *Amanita citrina* produces bufotenine [59,93,94,95]. The present study confirmed that one closely related species, *A*. *sinocitrina*, also produces bufotenine. Meanwhile, no tryptamine alkaloids were detected in the variety *A. citrina* var. *grisea*. *Amanita porphyria*, a similar species from sect. *Validae* which is closely related to *A. citrina* [39,40,98] (Figure 2), does not contain bufotenine but contains 5-hydroxytryptophan [94]. Among these >100 species, how many species are poisonous needs to be studied in the future.

## 4. Conclusions

This study demonstrated that *A*. *citrina* and *A*. *sinocitrina* contain bufotenine. To the best of our knowledge, this is the first study to report on bufotenine discovered from *A*. *sinocitrina*. Additional research is urgently required to determine the prevalence of bufotenine across various species. Future work should explore the biosynthetic pathways and potential pharmacological activities of bufotenine in other *Amanita* species.

## 5. Materials and Methods

### 5.1. Chemicals and Reagents

A series of certified reference standards, including muscarine, psilocybin, psilocin, bufotenine, ibotenic acid, muscimol, α-amanitin, β-amanitin, γ-amanitin, phalloidin, phallacidin and phallisacin, were acquired from Alta Scientific Co., Ltd. (Tianjin, China) in methanol solution and weresubsequently preserved at −20 °C in a freezer. The QuEChERS-PP purification column, incorporating multiwalled carbon nanotubes as the stationary phase, was supplied by Huaren Health Co., Ltd. (Zhengzhou, China). HPLC-grade solvents and reagents—acetonitrile, methanol, ammonium acetate and formic acid—were sourced from Merck Ltd. (Darmstadt, Germany). For all experimental procedures, ultrapure water (resistivity = 18.2 MΩ/cm, TOC < 3 ppb) was generated using a Milli-Q purification system (Millipore, Billerica, MA, USA).

### 5.2. Materials

Specimens were collected from Guizhou, Yunnan, Hunan and Hubei Provinces, China (Table 1). All fresh mushrooms were dried at 45 °C, and were then stored in the herbarium in the National Institute of Occupational Health and Poison Control, Chinese Center for Disease Control (NIOHP, China CDC). Toxin analysis was performed using 10 mg aliquots of the lyophilized fungal fruiting bodies.

### 5.3. Species Identification

Morphological identification was mainly performed as per the methods established in previous studies [18,19,24]. Internal transcribed spacer (ITS) was selected for phylogenetic analysis, and maximum parsimony (MP) analyses were applied to the ITS dataset [24,39,100].

### 5.4. Sample Preparation for Toxin Detection

A dried mushroom specimen (10 mg) was homogenized with 2 mL of a methanol–water mixture (70:30, *v*/*v*) in a 15 mL centrifuge tube using vortex agitation. The resulting suspension was subjected to ultrasonic extraction for 60 min, followed by centrifugation (15,000 rpm, 4 °C, 5 min). The clarified supernatant was then passed through a QuECHERS-PP column to eliminate matrix interferences. Subsequently, 10 μL of the purified extract was diluted with a methanol–water solution (5:95, *v*/*v*) to a total volume of 1 mL. Prior to UPLC–MS/MS analysis, the prepared solution was centrifuged at 21,000 rpm for 2 min to ensure clarity [101].

For the qualitative screening of mushroom toxins, 20 µL of mushroom extract was pipetted into a vial and diluted to 1 mL with an initial mobile phase (10% B: acetonitrile). The targeted screening of 12 mushroom toxins was performed under an optimal UPLC–MS/MS detection. The toxins detected in the samples were quantitatively analyzed.

In the quantitative analysis, *Lentinula edodes* were used as the matrix blank mushroom and spiked samples. The matrix blank mushroom and spiked samples were extracted using the same sample preparation method described above, followed by UPLC–MS/MS analysis. Serial concentrations of positive toxins were added to a matrix blank mushroom sample solution to establish the calibration curves for UPLC–MS/MS analysis.

### 5.5. LC–MS/MS Conditions

UPLC–MS/MS was carried out with a Waters ACQUITY I-Class UPLC system coupled with a Waters Xevo TQ-S MS/MS system (Waters, Milford, MA, USA). The mass spectrometry acquisition parameters of mushroom toxins and the specific parameters of UPLC were chosen according to our previous work [101]. Bufotenine and psilocin were isomers ([M + H]^+^ = 205.1) with the same mass spectrum parameters. The mass spectrum parameters of the 12 mushroom toxins are in Table 2.

Briefly, the conditions were as follows: the chromatographic separation was carried out using an Atlantis T3 analytical column (2.1 × 100 mm, 3 μm; Waters, USA) and the mobile phase consisted of two components: (A) an aqueous solution of ammonium acetate (10 mmol/L) and (B) acetonitrile, delivered at a constant flow rate of 0.3 mL/min. For mass spectrometric detection, an electrospray ionization (ESI) source was operated in positive-ion mode (ESI+). Data acquisition was conducted via multiple reaction monitoring (MRM). The ionization parameters were optimized as follows: the source temperature was maintained at 550 °C and the spray voltage was set to 5500 V.

## Figures and Tables

**Table 1:** The bufotenine contents and species identification information in different mushroom specimens used in the present study.

Species	Specimen Voucher	Collection Date	Location	ITS Numbers	Bufotenine (g/kg)
*A. citrina*	150913-24	13-Sep-2015	China: Guizhou	OR345188	2.90 ± 0.27
*A. citrina* var. *grisea*	WX20170922-067	22-Sep-2017	China: Yunnan	OR345187	<LOD
*A. sinocitrina*	141025-05	25-Oct-2014	China: Guizhou	-	2.70 ± 0.19
	150914-02	14-Sep-2015	China: Guizhou	OR345184	1.19 ± 0.02
	150914-18	14-Sep-2015	China: Guizhou	OR345177	3.42 ± 0.66
	150914-27	14-Sep-2015	China: Guizhou	MN647023	4.10 ± 0.95
	171012-19	12-Oct-2017	China: Guizhou	OR345179	4.00 ± 0.73
	171012-23	12-Oct-2017	China: Guizhou	OR345186	2.66 ± 0.52
	171012-38	12-Oct-2017	China: Guizhou	OR345178	6.70 ± 1.36
	DL20170920-011	20-Sep-2017	China: Guizhou	OR345185	1.22 ± 0.08
	GZHS20200703-04	03-Jul-2020	China: Guizhou	OR345182	2.97 ± 0.31
	GZHS20200703-06	03-Jul-2020	China: Guizhou	OR345183	4.48 ± 0.97
	GZRH20200701-04	01-Jul-2020	China: Guizhou	OR345174	4.20 ± 0.85
	GZRH20200709-02	09-Jul-2020	China: Guizhou	OR345180	3.85 ± 0.68
	GZSN20200617-006	17-Jun-2020	China: Guizhou	OR345175	2.88 ± 0.16
	HB20190708-02	08-Jul-2019	China: Hubei	OR345181	3.70 ± 0.69
	HNZJJCL20200624-02	24-Jun-2020	China: Hunan	OR345176	5.26 ± 1.15
	HNZJJCL20200924-06	24-Sep-2020	China: Hunan	OR345173	2.66 ± 0.65

**Table 2:** The mass spectrum parameters of 12 mushroom toxins.

Analyte	Precursor Ion (Q1, *m*/*z*)	Product Ion (Q3, *m*/*z*)	DP (V)	CE (V)	Retention Time (min)
α-amanitin	919.4	85.7 ^a^, 259.3 ^b^	85, 85	107, 60	1.92
β-amanitin	920.6	85.6 ^a^, 259.5 ^b^	103, 103	104, 59	1.53
γ-amanitin	903.6	86.1 ^a^, 243.1 ^b^	125, 125	123, 60	3.21
Phalloidin	789.7	157.2 ^a^, 85.7 ^b^	108, 108	108, 81	5.13
Phallacidin	847.4	157.2 ^a^, 330.3 ^b^	101, 101	80, 63	4.53
Phallisacin	863.6	156.8 ^a^, 85.8 ^b^	101, 101	117, 88	2.79
Muscarine	174.2	57.1 ^a^, 97.2 ^b^	73, 70	32, 26	1.30
Ibotenic acid	158.9	113.2 ^a^, 142.1 ^b^	50, 50	16, 17	0.87
Muscimol	115.1	98.0 ^a^, 68.1 ^b^	43, 40	17, 20	0.89
Psilocybin	285.2	58.1 ^a^, 205.3 ^b^	82, 82	53, 25	1.32
Bufotenine	205.2	58.1 ^a^, 160.1 ^b^	60, 69	33, 19	3.26
Psilocin	205.1	58.1 ^a^, 160.1 ^b^	40, 45	32, 23	3.51

Notions: ^a^ quantified ions; ^b^ qualitative ions.

## Data Availability

The original contributions presented in this study are included in the article. Further inquiries can be directed to the corresponding authors.
